# Implications of Brexit on the effectiveness of the UK soft drinks industry levy upon CHD in England: a modelling study

**DOI:** 10.1017/S1368980018002367

**Published:** 2018-10-09

**Authors:** Paraskevi Seferidi, Anthony A Laverty, Jonathan Pearson-Stuttard, Maria Guzman-Castillo, Brendan Collins, Simon Capewell, Martin O’Flaherty, Christopher Millett

**Affiliations:** 1 Public Health Policy Evaluation Unit, School of Public Health, Imperial College London, Reynolds Building, St Dunstan’s Road, London W6 8RP, UK; 2 Department of Public Health and Policy, University of Liverpool, Liverpool, UK

**Keywords:** Soft drinks levy, Sugar, CHD, Brexit, Inequalities

## Abstract

**Objective:**

An industry levy on sugar-sweetened beverages (SSB) was implemented in the UK in 2018. One year later, Brexit is likely to change the UK trade regime with potential implications for sugar price. We modelled the effect of potential changes in sugar price due to Brexit on SSB levy impacts upon CHD mortality and inequalities.

**Design:**

We modelled a baseline SSB levy scenario; an SSB levy under ‘soft’ Brexit, where the UK establishes a free trading agreement with the EU; and an SSB levy under ‘hard’ Brexit, in which World Trade Organization tariffs are applied. We used the previously validated IMPACT Food Policy model and probabilistic sensitivity analysis to estimate the effect of each scenario on CHD deaths prevented or postponed and life-years gained, stratified by age, sex and socio-economic circumstance, in 2021.

**Setting:**

England.

**Subjects:**

Adults aged 25 years or older.

**Results:**

The SSB levy was associated with approximately 370 (95 % uncertainty interval 220, 560) fewer CHD deaths and 4490 (2690, 6710) life-years gained in 2021. Associated reductions in CHD mortality were 4 and 8 % greater under ‘soft’ and ‘hard’ Brexit scenarios, respectively. The SSB levy was associated with approximately 110 (50, 190) fewer CHD deaths in the most deprived quintile compared with 60 (20, 100) in the most affluent, under ‘hard’ Brexit.

**Conclusions:**

Our study found the SSB levy resilient to potential effects of Brexit upon sugar price. Even under ‘hard’ Brexit, the SSB levy would yield benefits for CHD mortality and inequalities. Brexit negotiations should deliver a fiscal and regulatory environment which promotes population health.

Taxation has been recommended by the WHO as an effective measure to minimise intake of sugar-sweetened beverages (SSB)^(^ ^1^ ^)^. This is supported by recent evidence from Mexico and Berkeley showing that SSB sales and consumption decreased after SSB taxes were implemented, especially among populations of lower socio-economic circumstance (SEC)^(^ ^2^ ^–^ ^4^ ^)^. Such reductions can have significant health benefits to the population, given the abundance of evidence associating SSB intake with the risk of being overweight or obese^(^ ^5^ ^)^ and of developing diabetes^(^ ^6^ ^)^ and CVD^(^ ^7^ ^)^. For example, a modelling study suggested that the SSB tax in Mexico would reduce CVD deaths by 10 900 over the ten years following its implementation^(^ ^8^ ^)^.

The UK Government implemented an SSB industry levy in April 2018, as part of its Childhood Obesity Plan^(^ ^9^ ^)^. However, the effect of the SSB levy might be affected by the impending exit of the UK from the EU in March 2019, known as ‘Brexit’. Brexit is likely to result in a new trade regime, which could range from a free trade regime that eliminates all trade barriers to a default World Trade Organization (WTO) membership that enforces tariffs to all trade relationships.

The trade arrangements that the UK will adopt after Brexit are likely to influence the price of food and food ingredients including sugar^(^ ^10^ ^)^, which currently follows European regulations under the Common Agricultural Policy. This could potentially interfere with the effectiveness of the SSB levy in the UK. We aimed to estimate the implications of Brexit on the price of sugar and the impact of the implemented SSB levy on CHD mortality and inequalities in England.

## Methods

We modelled the potential effects of the SSB industry levy on SSB price and consumption and compared it with its effect under a ‘soft’ and a ‘hard’ Brexit scenario. We then estimated the effect of the SSB consumption changes on CHD mortality in England in 2021, stratified by age, sex and SEC.

### The IMPACT Food Policy model

We extended the previously validated IMPACT Food Policy model^(^ ^11^ ^–^ ^15^ ^)^ to estimate the effect of changes in the price of SSB on CHD mortality and life-years. The model has been validated among adults aged 25 years or above^(^ ^16^ ^)^ and translates the estimated changes in SSB price into changes in SSB intake, using price effect estimates, and subsequently into changes in CHD mortality, using appropriate relative risks. The policy impacts on CHD were estimated for 2021, the year when the UK plans to officially exit its Brexit transition period and initiate its new relationship with its trading partners. The model assumes a constant effect of the policies over time. All model assumptions are presented in the online supplementary material, Supplemental Table 1.

Changes in CHD mortality were expressed in CHD deaths prevented or postponed (DPP) and life-years gained (LYG). Details on the DPP calculation methodology are presented in the online supplementary material, Supplemental File 1. LYG were calculated as the product of DPP and median survival. A previous modelling study^(^ ^13^ ^)^ provided age-, sex- and SEC-specific median survival for three population subgroups (diagnosed CHD, undiagnosed CHD, no CHD) and the proportion of CHD deaths in each group.

### Data sources

An overview of the data sources used is presented in the online supplementary material, Supplemental Table 2. To estimate the effect of Brexit on price of sugar, we used 2015 sugar trade data from UK government sources^(^ ^17^ ^,^ ^18^ ^)^ and sugar price data from the EU^(^ ^19^ ^)^. Further details on trade and price data sources are provided in the online supplementary material.

We obtained CHD mortality (ICD-10 (International Statistical Classification of Diseases and Related Health Problems, 10th revision) codes: I20–I25) data projected to 2021 from Guzman Castillo *et al.* ^(^ ^20^ ^)^. Those researchers used a hierarchical Bayesian Age Period Cohort (BAPC) model to predict CHD mortality in England and Wales, taking previously declining trends into account. In order to estimate CHD mortality in England only, as well as SEC-stratified CHD mortality, we used 2013 mortality data available from the Office for National Statistics^(^ ^21^ ^)^. We assumed that differences in mortality between England and Wales and differences across SEC groups would remain unchanged over time. We stratified the mortality estimates by 10-year age groups (from 25–34 years to 85+ years) and sex. We defined SEC using the Index of Multiple Deprivation (IMD), a measure of deprivation in small areas in England (LSOA). For this model, we used quintiles of the 2010 IMD scores, with quintile 1 representing the most affluent areas and quintile 5 the most deprived.

We used results from a meta-analysis of interventional and prospective observational studies that assessed the effect of price change interventions on dietary intake^(^ ^22^ ^)^, in order to translate SSB price change into SSB consumption. That meta-analysis found that a 10 % increase in the price of SSB was associated with a 6·7 % decrease in their consumption over time. To consider different responses to the SSB price change among SEC groups, we adjusted price effect estimates for SEC using data from an observational study which evaluated the one-year effect of an SSB tax on SSB purchases among three SEC groups in Mexico^(^ ^2^ ^)^. The price effect estimates used in this model are shown in the online supplementary material, Supplemental Table 3.

We estimated SSB intake of the study population using data from the National Diet and Nutrition Survey (NDNS) rolling programme, years 1–4 (2008/09–2011/12)^(^ ^23^ ^)^. This is a nationally representative survey of UK children and adults randomly selected from a list of all UK addresses and uses four-day food diaries to estimate dietary intake. We calculated weighted mean intake by age (20-year age groups from 25–44 years to 65+years), sex and IMD quintile among the English population. Due to the small sample size, we combined IMD quintiles 1 and 2, and quintiles 4 and 5, thus calculating estimates for three SEC groups overall.

Finally, to estimate the effect of SSB intake on CHD, we used age-specific relative risks for CHD. Pooled analyses of cohort studies^(^ ^24^ ^)^ provided BMI-adjusted and BMI-mediated relative risks for incident CHD by SSB intake (number of servings). We expect that SSB intake would not have any additional effect on the proportion of CHD cases that are fatal, so we assumed a linear relationship between CHD risk and mortality. The overall CHD effect was estimated as the additive effect of a BMI-adjusted and a BMI-mediated effect^(^ ^24^ ^)^.

### Policy scenarios

We modelled three potential scenarios:

1. SSB industry levy;
2. SSB industry levy under a ‘soft’ Brexit; and
3. SSB industry levy under a ‘hard’ Brexit.

The SSB levy is a two-rate industry levy, depending on the sugar content of SSB. A levy of 18 pence (p) per litre is applied to SSB containing 5–8 g sugar/100 ml and 24 p per litre to SSB containing more than 8 g sugar/100 ml. Using a weighted average of SSB sales data from 2017^(^ ^25^ ^)^, we estimated an equivalent pooled single-rate levy.

The two Brexit scenarios assumed that the UK Government will leave the EU Single Market and Customs Union as per its publicly stated negotiating position in February 2017^(^ ^26^ ^)^ (Table 1). We hypothesised that under the ‘soft’ Brexit scenario the UK will establish a zero duty Free Trading Agreement for sugar with the EU but will trade under WTO regulations with third (non-EU) countries. This means that the UK would have to apply common tariffs to sugar imports from all third countries, including those currently under a Free Trading Agreement with the EU. The more impactful ‘hard’ Brexit scenario assumes that the UK will take a WTO default position, thus having no preferential trading agreements in place. In that case, the UK would apply WTO tariffs to sugar imports both from the EU and third countries. Under these scenarios, we expect changes in the price of sugar due to increased trade facilitation costs and changes in applied import tariffs. More details on the calculations to estimate the effect of Brexit on price of sugar are presented in the online supplementary material, Supplemental Table 4 and Supplemental File 1.

**Table 1:** UK trade policy before and after Brexit

UK trade policy before Brexit	As part of the EU, the UK abides by EU trade policy. This means that it is part of the EU Single Market and the Customs Union, being guaranteed free access to the EU market, benefiting from European Free Trading Agreements and applying European external tariffs when trading with third countries
UK trade policy after Brexit	In a White Paper published in February 2017^(^ ^26^ ^)^, the UK Government showed its intentions to leave the Single Market and the Customs Union and seek new preferential trading agreements with the EU and third countries

After estimating the effect of Brexit on the price of sugar, we calculated the potential increase in the cost of SSB, based on the amount of sugar needed for their production. Mean added sugar content of SSB in the UK was estimated using information from brands’ websites and UK sales data from Euromonitor International^(^ ^27^ ^)^.

We combined the SSB levy and Brexit effects to estimate the change in production costs of SSB in £/l, under each scenario. We then assumed that the industry will pass the cost increase to the consumer on different pass-through rates, which can vary depending on SSB type, retailer and package size^(^ ^3^ ^,^ ^28^ ^)^. An observational study in Mexico showed that price pass-through after implementation of an excise tax was 100 % overall, and varied between 36 and 150 % among different SSB products^(^ ^28^ ^)^. A similar study in Berkeley showed that one year after a tax implementation SSB prices were not significantly changed among some retailers but reached a 220 % pass-through rate among others^(^ ^3^ ^)^. In the present analysis, we considered three potential price pass-through rates: 80, 100 and 120 %. Finally, we estimated the relative effect of each scenario on SSB consumer price using mean SSB price estimates, calculated from expenditure and purchase data from the Living Costs and Food Survey (2014), a national representative survey of food consumption and expenditure in UK households^(^ ^29^ ^)^.

### Probabilistic sensitivity analysis

We performed a probabilistic sensitivity analysis to incorporate parameter uncertainty into the model. We used Monte Carlo simulation to repeatedly draw random values of model inputs from their respective statistical distributions. The model inputs that added uncertainty to the model were the sugar imports as a percentage of sugar supply, € to £ exchange rate, SSB price, price effect on consumption, mean SSB intake, relative risks, overweight prevalence, CHD mortality and median survival. The distributions used for each input are shown in the online supplementary material, Supplemental Table 5. We conservatively assumed that the model parameters included in the probabilistic sensitivity analysis were independent, when in fact some are likely to be correlated. This may overestimate the uncertainty of the model results. We used the Microsoft Excel add-in ‘Ersatz’ version 1.35 (EpiGear, Brisbane, Australia) to obtain 95 % uncertainty intervals (UI) from 10000 iterations.

## Results

### Effect of modelled scenarios on price of sugar and sugar-sweetened beverages

Imported sugar accounted for 64 % of the total UK sugar supply in 2015. Approximately half of sugar imports to the UK (53 %) came from the EU, while the rest were from third countries, mainly the African, Caribbean and Pacific States. The majority of the third country imports (94 %) were imported under a preferential agreement between the EU and third countries (see online supplementary material, Supplemental Table 6).

We estimated that a ‘soft’ and a ‘hard’ Brexit will increase price of sugar by 92 and 203£/t, respectively. With an estimated sugar content of SSB in the UK market at 93g/l, we calculated an increase in SSB production costs of 1 and 2p/l under ‘soft’ and ‘hard’ Brexit scenarios, respectively. The SSB levy would cost the industry 23p/l on average. The effect of each scenario on final SSB consumer price under different pass-through rates is shown in Table 2.

**Table 2:** Effect of each scenario on the final sugar-sweetened beverage (SSB) price

Scenario	Price pass-through (%)	Change in final SSB price (£/l)	Change in final SSB price* (%)
Scenario 1: SSB industry levy			
Scenario 1a	80	0·19	30
Scenario 1b	100	0·23	38
Scenario 1c	120	0·28	46
Scenario 2: SSB industry levy under ‘soft’ Brexit			
Scenario 2a	80	0·19	32
Scenario 2b	100	0·24	39
Scenario 2c	120	0·29	47
Scenario 3: SSB industry levy under ‘hard’ Brexit			
Scenario 3a	80	0·20	33
Scenario 3b	100	0·25	41
Scenario 3c	120	0·30	49

* Assumes mean SSB price of 0·61£/l, estimated using data from the Living Costs and Food Survey (2014)^(^ ^29^ ^)^.

### Effect of modelled scenarios on intake of sugar-sweetened beverages

Mean baseline SSB intake in the English population aged 25 years or above was 99·5 g/d. It varied across age and sex groups, with younger men having the higher intake across all SEC categories. The most affluent group had the lowest SSB intake. Weighted means of SSB intake by age, sex and SEC group are presented in the online supplementary material, Supplemental Table 7.

We estimated that the SSB levy would reduce SSB intake in the overall population by approximately between 21 and 31 % in 2021, depending on the pass-through rate. This estimated reduction varied among SEC groups, under both Brexit scenarios (online supplementary material, Supplemental Table 8). For example, SSB intake in the most affluent group might decrease by approximately 23 % after the levy implementation, compared with 38 % in the most deprived. Under a ‘hard’ Brexit scenario, the levy could reduce SSB intake by approximately 25 % in the low and middle deprivation groups, compared with 41 % in the most deprived group.

### Effect of modelled scenarios on CHD mortality

CHD deaths in 2021 were projected to be approximately 38000 (95 % CI 30600, 46500). CHD mortality was higher among the more deprived populations. Detailed numbers of deaths per each group are presented in the online supplementary material, Supplemental Table 9.

**Fig. 1:**
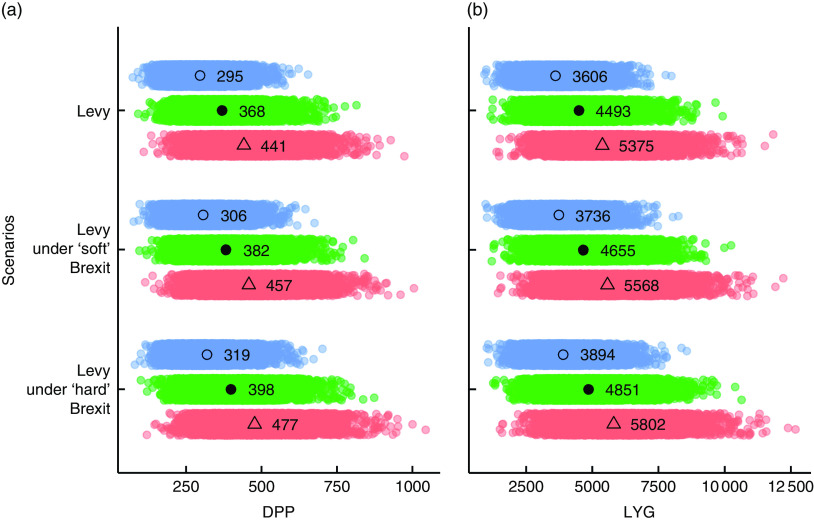
(colour online) Effect of each scenario on the effectiveness of the UK soft drinks industry levy upon CHD in the English population aged 25 years or older, in 2021: (a) CHD deaths prevented or postponed (DPP); (b) CHD life-years gained (LYG). (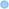, 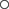), 80 % price pass-through; (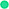, 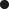), 100 % price pass-through; ( 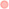, 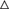 ), 120 % price pass-through. Each point ( 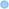 ,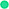 , 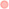 ) represents the DPP and LYG estimated from one iteration of the probabilistic analysis. The mean of 10 000 iterations is noted for each scenario

The CHD DPP and LYG estimates for each policy scenario are presented in Fig. 1. In the case of a complete pass-through of the cost increase to the consumer, the SSB levy might save approximately 370 (95 % UI 220, 560) CHD deaths and 4490 (95 % UI 2690, 6710) life-years in 2021. A ‘soft’ Brexit scenario will save 4 % more CHD deaths (approx. 10 deaths) and life-years (approx. 160 life-years) and a ‘hard’ Brexit scenario 8 % more CHD deaths (approx. 30 deaths) and life-years (approx. 360 life-years) compared with an SSB levy only scenario. The industry response to the cost increase would substantially influence the effectiveness of the policies, as an 80 % pass-through could save 20 % fewer CHD deaths and life-years across all scenarios and a 120 % pass-through 20 % more, compared with a complete price pass-through to the consumer.

**Fig. 2:**
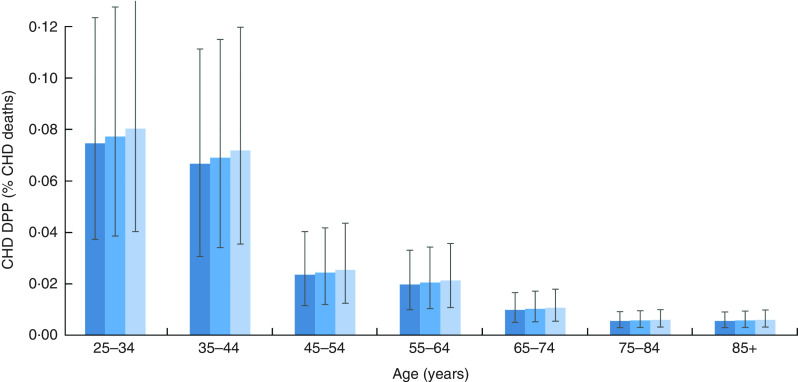
(colour online) Age differential effect of each scenario (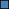 , levy; 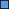, levy under ‘soft’ Brexit; 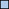, levy under ‘hard’ Brexit), in the case of 100 % price pass-through, on the effectiveness of the UK soft drinks industry levy upon CHD deaths prevented or postponed (DPP), expressed as a percentage of baseline CHD deaths per age group, in the English population aged 25 years or older, in 2021. The vertical bars are 95 % uncertainty intervals

People of younger age would benefit the most from the SSB levy under all Brexit scenarios (Fig. 2). The levy might reduce CHD deaths in 2021 by approximately 7 % among people aged 25–34 years and 35–44 years but by only 1 % among people over 65 years old.

**Fig. 3:**
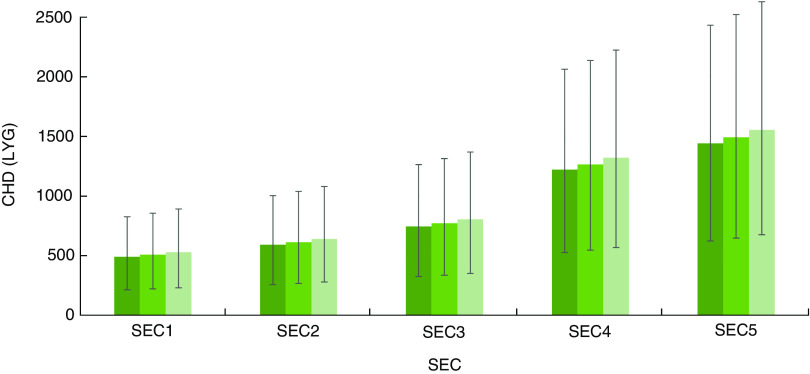
(colour online) Socio-economic circumstance (SEC) differential effect of each scenario ( 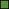 , levy; 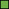 , levy under ‘soft’ Brexit; 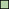 , levy under ‘hard’ Brexit), in the case of 100 % price pass-through, on the effectiveness of the UK soft drinks industry levy upon CHD in terms of life-years gained (LYG) in the English population aged 25 years or older, in 2021. SEC1 is the most affluent group and SEC5 the most deprived. The vertical bars are 95 % uncertainty intervals

Changes in SSB intake under all scenarios would particularly benefit people of lower SEC. Estimated DPP in the most deprived group were almost twofold higher than the DPP in the most affluent (Table 3). The levy would also lead to approximately 1560 (95 % UI 680, 2630) LYG in the most deprived group and just 530 (95 % UI 230, 890) LYG in the most affluent, under a ‘hard’ Brexit scenario (Fig. 3).

**Table 3:** Effect of each scenario on the effectiveness of the UK soft drinks industry levy upon CHD deaths prevented or postponed (DPP) in the English population aged 25 years or older, stratified by Index of Multiple Deprivation (IMD) quintile, in 2021

	SSB industry levy		SSB industry levy under ‘soft’ Brexit		SSB industry levy under ‘hard’ Brexit	
	DPP	95 % UI	DPP	95 % UI	DPP	95 % UI
80 % pass-through						
1st IMD quintile (most affluent)	41	17, 72	43	18, 74	44	19, 77
2nd IMD quintile	49	21, 85	50	21, 88	53	22, 92
3rd IMD quintile	52	22, 90	54	23, 93	56	24, 97
4th IMD quintile	72	30, 124	75	32, 129	78	33, 134
5th IMD quintile (most deprived)	81	35, 139	84	36, 144	88	37, 150
Total	295	176, 446	306	183, 461	319	190, 481
100 % pass-through						
1st IMD quintile (most affluent)	51	22, 89	53	23, 93	56	23, 97
2nd IMD quintile	61	26, 106	63	27, 110	66	28, 114
3rd IMD quintile	65	27, 112	67	28, 116	70	30, 121
4th IMD quintile	90	38, 155	93	39, 160	97	41, 167
5th IMD quintile (most deprived)	101	43, 173	105	45, 179	109	47, 186
Total	368	220, 556	382	228, 575	398	237, 600
120 % pass-through						
1st IMD quintile (most affluent)	62	26, 107	64	27, 111	67	28, 116
2nd IMD quintile	73	31, 126	75	32, 131	79	33, 137
3rd IMD quintile	78	33, 135	81	34, 139	84	36, 145
4th IMD quintile	108	46, 185	112	47, 192	117	49, 199
5th IMD quintile (most deprived)	121	52, 207	126	54, 214	131	56, 223
Total	441	264, 666	457	273, 689	477	285, 718

UI, uncertainty interval.

## Discussion

The present study estimated the potential implications of Brexit on the price of sugar and consequent impacts of the proposed SSB levy on CHD mortality and inequalities in England. The SSB levy may increase SSB prices by approximately 38 % leading to an estimated 26 % decrease in SSB intake. This would prevent approximately 370 CHD deaths and generate approximately 4490 life-years in 2021. The SSB levy was associated with additional reductions in CHD mortality of 4 and 8 % under ‘soft’ and ‘hard’ Brexit scenarios, respectively. The SSB levy would particularly benefit people in more deprived groups under both Brexit scenarios. This is attributed to the higher CHD mortality rates, higher SSB intake and higher sensitivity to SSB price changes among the more deprived. The persisting inequalities in CHD mortality in England despite rapid reductions at aggregate level^(^ ^30^ ^)^ highlight the importance of this finding.

The impact of trade regimes on health and national public health policies that aim to tackle diet-related chronic diseases remains under-investigated^(^ ^31^ ^)^. The present study is the first to quantify the potential impacts of Brexit on the SSB levy effectiveness through changes in the UK trade policy. Previous studies have suggested an association between trade agreements and SSB sales and intake. For example, a longitudinal analysis of forty-four low- and middle-income countries showed significant associations between tariffs on SSB and per capita imports and sales^(^ ^32^ ^)^. A systematic review identified an increase in sales and consumption of SSB in low- and middle-income countries after entering a trade agreement^(^ ^31^ ^)^. While the UK already has an established SSB industry operating domestically, SSB production relies heavily on sugar imports. Thus, changes in the trade regime are more likely to affect the UK SSB market indirectly through changes in the price of sugar, as modelled in the current analysis.

Our findings are reassuringly consistent with studies elsewhere. A quasi-experimental study concluded that abolition of import tariffs on beverage syrups, including high-fructose corn syrup, in Canada was significantly associated with an increase in their per capita energy supply^(^ ^33^ ^)^. However, the researchers did not investigate the consequent effect on beverages that incorporate high-fructose corn syrup, like SSB. A modelling study in France suggested that a decrease in the price of sugar by 228€/t, due to changes in the EU Common Agricultural Policy, could decrease SSB prices on average by 0·02€/l, with high variation among different SSB brands^(^ ^34^ ^)^. These findings are consistent with our analysis which suggested that a sugar price reduction of 92 and 203£/t under a ‘soft’ and a ‘hard’ Brexit scenario would result in changes in the price of SSB of approximately 0·01 and 0·02£/l, respectively.

We estimated an SSB price increase of approximately 30–46 % due to the SSB levy, depending on the price pass-through to the consumer. This is higher than the SSB levy effect estimated in previous studies in other locations, due to differences in the implemented tax rates. For example, in Mexico, the applied excise tax represented approximately a 10 % increase in SSB price^(^ ^28^ ^)^ and in Berkeley approximately 15 %^(^ ^3^ ^)^. However, we applied pass-through rates as observed in these jurisdictions. Variations of pass-through rates between types of SSB and points of sale were not taken into account, as our analysis investigated the SSB market overall.

Our analysis estimated that the SSB intake reduction due to the levy was associated with approximately 370 fewer deaths or a 1 % reduction in estimated CHD mortality. A similar analysis in the USA found that a 10 % SSB tax will result in approximately 0·4 % reduction in CHD mortality^(^ ^12^ ^)^ over 15 years, while a modelling study in Mexico showed that a 20 % reduction in SSB intake will result in approximately 2 % reduction in CHD mortality over a period of 10 years^(^ ^8^ ^)^. These results are comparable to our one-year analysis as the authors assumed a sustained effect throughout the predicting period. The variation in the estimated results can be attributed in part to differences in the population intake of SSB and the magnitude of modelled interventions, as implemented SSB tax rates can vary significantly in different interventions.

The current analysis has a number of strengths. We used a previously validated food policy model^(^ ^11^ ^–^ ^15^ ^)^ which combines high-quality data, including detailed information on UK sugar trade, mortality projections that take declining trends in CHD mortality into account^(^ ^20^ ^)^ and nationally representative SSB intake data. It also enabled differential policy impacts between SEC groups to be modelled. Finally, uncertainty of inputs was incorporated into the model, using probabilistic sensitivity analysis.

However, there are some limitations that should also be considered. First, we used 2015 trade data to approximate sugar imports in 2021. We also used 2013 mortality and population data to adjust 2021 projections and assumed that CHD mortality and population differences between England and Wales and between SEC groups remained unchanged. The BAPC model also assumes that the age, period and cohort effects on mortality remain unchanged into the future, although it had the best predicted performance compared with conventional projections when validated^(^ ^20^ ^)^. We assumed an immediate effect of SSB intake on CHD, which might not be accurate. Moreover, we varied price effect estimates across SEC groups using SEC differential SSB purchase data after an SSB tax in Mexico^(^ ^2^ ^)^. Those researchers found that the most deprived group was 65 % more responsive to price change compared with the most affluent. Discrepancies in the inequality gap between Mexico and the UK might have overestimated the SEC differences in the current analysis; average real household net disposable income in the UK is roughly twice that of Mexico^(^ ^35^ ^)^. This model estimated the effects of the different scenarios on CVD only through CHD. Some evidence supports an SSB effect on stroke, mediated through BMI, mainly in overweight populations^(^ ^24^ ^)^. However, we conservatively excluded SSB effects on stroke from the model. Finally, we modelled three different pass-through rates to investigate the industry response to the SSB levy and Brexit effects, based on industry responses to SSB taxes and sugar price increases elsewhere. However, the industry might have additional responses to the levy, such as product reformulation and shifts in the market shares of mid- and high-sugar SSB^(^ ^36^ ^)^. For the current analysis, we estimated a single pooled levy rate based on the market distribution before the levy implementation, assuming it will stay unchanged until 2021.

The model did not directly account for substitutability of SSB with other foods or beverages as has been conducted in previous work^(^ ^37^ ^)^. This is because the estimates of price responsiveness used in our study were drawn from a meta-analysis of longitudinal studies which reflects actual dietary changes after a change in the price of SSB, accounting for substitutability and complementarity in real-world settings. Another advantage of these estimates is that they specifically investigate the effect of a price increase of SSB taking account of the direction of the price change. However, they did not account for the potential effect of the change in SSB price on the intake of other beverages. Observational data from Mexico^(^ ^2^ ^)^ suggest that the increase in untaxed beverages that followed the SSB tax implementation was mainly driven by an increase in bottled water, which is not likely to have significant effects on health outcomes. Finally, any unmeasured effects due to changes in the intake of other foods and beverages are likely to be consistent across the different scenarios modelled. Thus, we assumed that taking account of these effects would not significantly alter our conclusions.

The present study provides a first estimation of the potential effect of Brexit on the SSB industry levy in the UK and represents an important example for future research in this area. We conservatively quantified the potential effect of Brexit on the price of sugar only one year after the post-Brexit transitional period due to the complexities and uncertainties following Brexit in the medium and long terms. For example, the UK is unlikely to conclude preferential trading agreements with third countries immediately after the two-year transitional period, due to the extensive time that these negotiations commonly take to complete. However, in the long term, the UK could establish preferential trading agreements with competitive partners, such as Australia and Brazil^(^ ^10^ ^)^, which would allow an inflow of cheap sugar into the UK market. These changes are attractive to UK lobbying groups, such as the sugarcane refining industry, parts of which have been vocally in favour of Brexit^(^ ^38^ ^)^. Also, in the long term, the SSB industry might decide to shift towards domestically produced sugar, encouraging British farmers to increase their production to meet demand. In this model, we assumed that industry will maintain the current market split between imported and domestically produced sugar, given the time needed to achieve such a change.

Further studies could also evaluate potential effects of Brexit on the industry response to the SSB levy. We found that Brexit will probably cost the industry millions of pounds in ingredient expenses that might encourage product reformulation with reduction of sugar content in SSB. A previous investigation of industry responses to the UK SSB levy suggested that product reformulation could be the most beneficial result in terms of improving health outcomes^(^ ^36^ ^)^. However, Brexit might allow the industry to shift towards alternative sweeteners, like high-fructose corn syrup. This would be highly affected by the post-Brexit agricultural regime. Currently, the Common Agricultural Policy is in place for all EU countries, including the UK. Under the Common Agricultural Policy, sugar price was kept high using production quotas and minimum price guarantees, while production of high-fructose corn syrup was restricted, accounting for 3·5 % of the EU sweetener market^(^ ^19^ ^)^. The EU liberalised its sugar agricultural regime in October 2017 by removing these restrictions. This is likely to affect price and availability of sugar and high-fructose corn syrup and encourage their incorporation in SSB^(^ ^39^ ^)^. Should the UK adopt a similar liberalised agricultural regime for sugar post-Brexit, the costs for the SSB industry might drop, making SSB production more profitable and allowing the industry to resist public health initiatives, like the SSB industry levy.

Brexit may also have wider implications on the SSB industry and prices, which go beyond the price of sugar. For example, Brexit is likely to affect the UK food system as a whole, causing disruptions across the whole supply chain^(^ ^40^ ^)^. Changes in costs of packaging, distribution and retailing of SSB may further affect SSB consumer prices. Moreover, the food and beverage industry in the UK relies heavily on the EU workforce, with almost a third of its workers being EU citizens^(^ ^41^ ^)^. Restrictions in the movement of labour between the UK and the EU might burden the SSB industry with a significant labour shortage. Lastly, the SSB industry will not be exempted from the overall macroeconomic effects of Brexit in the UK economy, both in the short term^(^ ^42^ ^)^ and the long term^(^ ^43^ ^)^.

Finally, the significant increases in sugar price due to Brexit estimated in the current analysis may raise concerns about the effect of Brexit on other food commodities. For example, the UK is dependent on EU and third country imports for its fruit and vegetable supply^(^ ^18^ ^)^. Steep increases in fruit and vegetable prices post-Brexit might reduce their consumption with detrimental implications to public health, especially among low-income populations.

## Conclusion

Our study suggests that the UK SSB industry levy is likely to be resilient to potential Brexit effects on sugar price due to changes in the UK trade regime, even if trade occurs under WTO regulations. It also suggests that even under alternative Brexit scenarios the SSB levy is likely to remain progressive in terms of CHD inequalities. Brexit presents a crucial opportunity to achieve a healthier food system in the UK if negotiations deliver a fiscal and regulatory environment which promotes population health.

## Supplementary material

For supplementary material accompanying this paper visit https://doi.org/10.1017/S1368980018002367. click here to view supplementary material
