# Supplementary material for: Implications of Brexit on the effectiveness of the UK soft drinks industry levy upon CHD in England: a modelling study
Source: Public Health Nutr. 2018 Oct 9;21(18):3431–9. doi: 10.1017/S1368980018002367 (PMC10260949; doi:10.1017/S1368980018002367)
Supplement: Supplementary file 1 [file S1368980018002367sup001.docx]

**Supplementary Appendix**

*Estimating DPPs with the IMPACT Food Policy model*

The effect of the different scenarios on CHD were measured in deaths prevented or postponed (DPPs) stratified by age, sex, and socioeconomic circumstance (SEC). DPPs in each age-sex-SEC group were calculated using the following formula:

$(1-e^{beta\times SSBchange})\times deaths$,

where SSB change was the age-sex-SEC specific change in SSB intake due to the policy, and deaths were the CHD deaths in 2021 for each age-sex-SEC group. For the BMI-adjusted effect, beta was the natural logarithm of the age specific relative risk for incident CHD per serving of SSB. For the BMI-mediated effect, beta was the natural logarithm of the age specific relative risk for incident CHD per BMI unit multiplied by the effect estimate of SSB intake on BMI. This estimate was adjusted for overweight prevalence of the population, using weighted prevalence estimates from the Health Survey for England 2014^(1)^, to account for the different effect magnitude between overweight and non-overweight individuals.

*Trade data sources*

We used sugar import data from 2015, available from the HM Revenue & Customs (HMRC)^(2)^ and the Department for Environment Food & Rural Affairs (DEFRA),^(3)^ to estimate the effect of the Brexit scenarios on price of sugar. We obtained information on EU tariffs and trading agreements from the World Trade Organisation (WTO)^(4)^ and the UK government Trade Tariff service.^(5)^ We acquired a projected value for sugar price in 2021 from the European Commission^(6)^ in €/t which we translated into £/t using the annual average exchange rate in 2016.^(2)^

*Effect of the Brexit scenarios on price of sugar*

We assumed two potential Brexit scenarios, a “soft” Brexit and a “hard” Brexit. Under these two scenarios, changes in the price of sugar would be driven by two main factors: (1) increased facilitation costs to imports from the EU and (2) applied import tariffs. Increased trade facilitation costs will mostly be due to increased border controls in order to apply rules of origin, in both Brexit scenarios, and to further investigate EU regulation applications, in the “hard” Brexit scenario^(7)^. We hypothesised them at 5% and 8% respectively, based on a previous modelling analysis^(8)^. In both Brexit scenarios, tariffs will increase the price of imports from third countries that were previously under an FTA with the EU, while in the “hard” Brexit scenario, further increases will also occur due to tariffs applied to EU imports. We assumed that these tariffs would be equal to the EU import tariffs currently in place, which are 33.9 €/t and 41.9 €/t depending on the type of sugar imported. The Appendix table 4 summarises the changes in the price of sugar under the two Brexit scenarios.

**Supplementary Appendix – Tables**

Appendix Table 1. Model assumptions

| ***Policy scenarios*** |
| --- |
| The industry passes the cost increase to the consumer. |
| The proportion of SSBs with sugar content 5-8 g/100 mL and SSBs with sugar content >8 g/100 mL in the SSB market in 2021 is approximated using 2016 sales data^(9)^. |
| The ratio of imported and domestically produced sugar used by the SSB industry is the same as their ratio in the overall market in 2021 and is approximated using 2015 data. |
| The difference in responsiveness to price among socioeconomic groups in Mexico is the same as in the UK. |
| ***Effects on mortality*** |
| There is an immediate effect of reducing SSB consumption on CHD mortality. |
| The differential mortality between England and Wales and between socioeconomic groups in 2013 is sustained in 2021. |
| There is a linear association between CHD risk and CHD mortality. |
| The Bayesian Age-Period-Cohort model used to project mortality to 2021 assumes that the observed age, period, and cohort effects on mortality remain unchanged into the future. |

Appendix Table 2. Data Sources for all model inputs

| Model input | Source |
| --- | --- |
| Sugar imports to the UK by sugar type and country of import | HM Revenue and Customs^(2)^ |
| Countries with a FTA with the EU | Gov.uk^(5)^ |
| Sugar imports in the UK as percentage of total sugar supply | Department for Environment, Food & Rural Affairs^(3)^ |
| EU Most-Favoured Nation tariff for sugar | World Trade Organisation^(4)^ |
| EU sugar price, prediction for 2021 | European Commission^(6)^ |
| € to £ exchange rate | HM Revenue and Customs^(2)^ |
| SSB sales by brand | Euromonitor International^(10)^ |
| Mean SSB sugar content by brand | brand websites |
| SSB industry levy rate | Office for Budget Responsibility^(11)^ |
| Market share of mid-sugar and high-sugar SSBs | British Soft Drinks Association, 2017^(9)^ |
| CHD mortality predictions for 2021 | Guzman-Castillo, 2014^(12)^ |
| CHD mortality in 2013 by country, age, sex, and IMD | Office for National Statistics^(13,14)^ |
| SSB purchase and expenditure (2015) | Family Food datasets, 2017^(15)^; provide aggregated data from the Living Costs and Food Survey, 2015 |
| Price change effect on consumption | Afshin, 2017^(16)^ |
| Price change effect on consumption by SEC | Colchero, 2016^(17)^ |
| Weighted SSB intake by age, sex, and IMD (1/2, 3, 4/5) | National Diet and Nutrition Survey Rolling Programme Years 1-4^(18)^ |
| RR for CHD per SSB serving by age | Micha, 2017^(19)^ |
| RR for CHD per 5 kg/m^2^ increase in BMI by age | Micha. 2017^(19)^ |
| BMI change (kg/m^2^) per SSB serving/d by BMI | Micha, 2017^(19)^ |
| Overweight and obesity prevalence | Health Survey for England 2014^(1)^ |
| Median survival (years) by age, sex, IMD for each of three CHD disease states and proportion of CHD deaths in each | Allen, 2015^(20)^ |

FTA, Free Trading Agreement; SSB, sugar-sweetened beverage; CHD, coronary heart disease; IMD, Index of Multiple Deprivation

Appendix Table 3. Effect of 1% change in SSB price on SSB intake overall, obtained from Afhsin et al,^(16)^ and adjusted by socioeconomic circumstance using information from Colchero et al.^(17)^

| Overall | Most affluent | Middle | Most deprived |
| --- | --- | --- | --- |
| -0.67 (-1.0, -0.31) | -0.61 (-0.94, -0.28) | -0.62 (-0.95, -0.28) | -1.01 (-1.55, -0.46) |

SSB. Sugar-sweetened beverage

Appendix Table 4. Added costs to sugar imports in each Brexit scenario

|  | EU imports of sugar | | | Third country imports of sugar under preferential arrangements | |
| --- | --- | --- | --- | --- | --- |
|  | Unrefined sugar imports | Refined sugar imports | | Unrefined sugar imports | Refined sugar imports |
| “Soft” Brexit | +5% of EU sugar price for facilitation costs increase | | | Tariff of 33.9 €/100 Kg applied | Tariff of 41.9 €/100 Kg applied |
| “Hard” Brexit | +8% of EU sugar price for facilitation costs increase | | | Tariff of 33.9 €/100 Kg applied | Tariff of 41.9 €/100 Kg applied |
|  | Tariff of 33.9 €/100 Kg applied | | Tariff of 41.9 €/100 Kg applied |  |  |

Appendix Table 5. Parameters and corresponding statistical distribution for model inputs used in the probabilistic sensitivity analysis

| Inputs | Distribution | Parameters | Source |
| --- | --- | --- | --- |
| Sugar Imports as % of total UK supply | Normal | Mean and SE estimated from import data between 2008 and 2016 | Department for Environment, Food & Rural Affairs^(3)^ |
| Exchange Rate | Beta | α1, α2 calculated from mean and standard deviation of monthly exchange rates in 2016 | HM Revenue and Customs^(2)^ |
| SSB price | Normal | Mean: SSB price in 2015  SE: assumed at 10% of the mean (no published uncertainty estimates available) | Family Food datasets, 2017^(15)^ |
| Price effect on consumption | Normal | Effect estimate and SE estimated from 95% CI, by SEC | Afshin, 2017^(16)^ |
| Mean SSB intake  (weighted) | Normal* | Weighted means and SEs, by age, sex, and SEC | National Diet and Nutrition Survey Rolling Programme Years 1-4^(18)^ |
| BMI-adjusted RR for CHD per SSB serving | Lognormal | RR and SE(lnRR) estimated from 95% CI, by age | Micha, 2017^(19)^ |
| RR for CHD per 5 kg/m2 increase in BMI | Lognormal | RR and SE(lnRR) estimated from 95% CI, by age | Micha, 2017^(19)^ |
| BMI change (kg/m2) per SSB serving/d | Lognormal | RR and SE(lnRR) estimated from 95% CI, by BMI categories | Micha, 2017^(19)^ |
| Overweight prevalence (weighted) | Binomial | Prevalence and N, by age and sex | Health Survey for England, 2014^(1)^ |
| CHD deaths 2021, projected using a BAPC model† | Pert | m: death projections, best estimate  a: death projections, lower 95% confidence limit  b: death projections, upper 95% confidence limit  by age, sex, and SEC | Guzman Castillo, 2014^(12)^ |
| Median Survival | Pert | m: median survival estimate  a: median survival estimate -20%  b: median survival estimate +20%  by CHD population subgroup, age, sex, and SEC | Allen, 2015^(20)^ |

SE, Standard Error; SSB, Sugar-sweetened beverage; CHD, Coronary Heart Disease; SEC, Socioeconomic Circumstance; RR, Relative Risk; BAPC, Bayesian Age Period Cohort

*According to the Central Limit Theorem, the means follow a normal distribution regardless of the distribution of the individual data. To calculate SEs of the weighted means, we centred strata with one sample unit at the grand mean instead of the stratum mean.

†When CHD deaths were stratified by IMD, there were two age/sex/IMD groups (females 25-34 in IMD=1 and IMD=2) that had zero deaths. In order to fit the Pert distribution, we replaced the zero deaths with one death in each subgroup.

Appendix Table 6. Sugar imports to the UK, 2015

| Input - Description | Unit | Percentage |
| --- | --- | --- |
| Sugar imports to the UK, 2015* | % of total UK supply | 64% |
| Sugar (unrefined) imports to the UK from the EU, 2015† | % of total UK imports | 3% |
| Sugar (refined) imports to the UK from the EU, 2015† | % of total UK imports | 50% |
| Zero-tariff sugar (unrefined) imports to the UK from third countries, 2015† | % of total UK imports | 34% |
| Zero-tariff sugar (refined) imports to the UK from third countries, 2015† | % of total UK imports | 10% |
| Sugar imports to the UK from third countries with applied tariffs, 2015† | % of total UK imports | 3% |

*By volume

†By value

Appendix Table 7. SSB intake by age, sex and IMD quintile, expressed as weighted means (SE) in grams/day from the National Diet and Nutrition Survey Rolling Programme Years 1-4^(18)^. IMDQ1/2 is the most affluent group and IMDQ4/5 the most deprived.

| Age/Sex group | Overall | | | IMDQ1/2 | | | IMDQ3 | | | IMDQ4/5 | | |
| --- | --- | --- | --- | --- | --- | --- | --- | --- | --- | --- | --- | --- |
|  | Mean | SE | N | Mean | SE | N | Mean | SE | N | Mean | SE | N |
| Men 25-44 | 185.8 | 15.8 | 263 | 169 | 17.9 | 112 | 214 | 33.8 | 51 | 189.4 | 25.8 | 100 |
| Men 45-64 | 80.3 | 10.7 | 285 | 83.2 | 14.2 | 130 | 81.5 | 15.0 | 53 | 75.8 | 15.2 | 102 |
| Men 65+ | 56.9 | 9.0 | 161 | 68.4 | 12.6 | 92 | 56.2 | 10.5 | 20 | 34.3 | 9.3 | 49 |
| Women 25-44 | 112 | 13.2 | 347 | 99.3 | 16.5 | 126 | 148 | 39.8 | 71 | 105.2 | 16.7 | 150 |
| Women 45-64 | 69.3 | 7.8 | 363 | 60 | 8.4 | 167 | 88.6 | 13.8 | 61 | 72.5 | 10.1 | 135 |
| Women 65+ | 55.5 | 7.1 | 186 | 56 | 9.1 | 100 | 57.2 | 12.4 | 41 | 52.5 | 12.0 | 45 |
| *Total* | *99.5* | *5.2* | *1605* | *90.5* | *5.8* | *727* | *119.7* | *14.1* | *297* | *100.6* | *7.3* | *581* |

SSB, Sugar-sweetened beverage; IMD, Index of Multiple Deprivation; SE, Standard Error; IMDQ, Index of Multiple Deprivation Quintile

*Mean intake in grams per day was translated in servings per day assuming 1 serving is 236.5 g (8oz)^(19)^

Appendix Table 8. Effect of each modelled scenario on SSB intake, overall and stratified by socio-economic circumstance

| Scenario | Overall | Most affluent | Middle | Most deprived |
| --- | --- | --- | --- | --- |
| Scenario 1: SSB industry levy |  |  |  |  |
| Scenario 1a: 80% pass-through | -21% | -19% | -19% | -31% |
| Scenario 1b: 100% pass-through | -26% | -23% | -24% | -38% |
| Scenario 1c: 120% pass-through | -31% | -28% | -28% | -46% |
| Scenario 2: SSB industry levy under “soft” Brexit |  |  |  |  |
| Scenario 2a: 80% pass-through | -21% | -19% | -20% | -32% |
| Scenario 2b: 100% pass-through | -27% | -24% | -24% | -40% |
| Scenario 2c: 120% pass-through | -32% | -29% | -29% | -48% |
| Scenario 3: SSB industry levy under “hard” Brexit |  |  |  |  |
| Scenario 3a: 80% pass-through | -22% | -20% | -20% | -33% |
| Scenario 3b: 100% pass-through | -28% | -25% | -25% | -41% |
| Scenario 3c: 120% pass-through | -33% | -30% | -31% | -50% |

SSB, Sugar-sweetened beverage;

Appendix Table 9. Number of CHD deaths (95% credible interval) in 2021 by age, sex, and IMD quintile, estimated using a Bayesian Age Period Cohort model (BAPC)^(12)^ and adjusted using 2013 CHD mortality data^(13)^. IMDQ1 is the most affluent group and IMDQ5 the most deprived.

| Age/Sex group | Overall | IMDQ 1 | IMDQ 2 | IMDQ3 | IMDQ4 | IMDQ5 |
| --- | --- | --- | --- | --- | --- | --- |
| Men 25-34 | 67 (50,92) | 9 (6,12) | 5 (4,7) | 17 (13,24) | 18 (14,25) | 17 (13,24) |
| Men 35-44 | 352 (263,483) | 35 (26,48) | 54 (40,74) | 58 (43,79) | 93 (70,128) | 112 (84,154) |
| Men 45-54 | 1228 (1006,1491) | 130 (107,158) | 152 (125,185) | 235 (193,286) | 306 (251,372) | 404 (331,491) |
| Men 55-64 | 2720 (2240,3261) | 344 (283,412) | 416 (343,499) | 522 (430,626) | 625 (514,749) | 813 (670,975) |
| Men 65-74 | 4923 (4043,5910) | 735 (603,882) | 925 (760,1110) | 981 (806,1178) | 1075 (883,1290) | 1207 (992,1449) |
| Men 75-84 | 7441 (6151,8907) | 1350 (1116,1616) | 1577 (1304,1888) | 1596 (1320,1911) | 1480 (1223,1771) | 1438 (1189,1721) |
| Men 85+ | 7807 (6436,9329) | 1679 (1384,2006) | 1797 (1482,2148) | 1739 (1434,2079) | 1448 (1194,1730) | 1143 (943,1366) |
| Women 25-34 | 11 (7,16) | 0 (0,0)* | 0 (0,0)* | 1 (0,1) | 5 (3,7) | 5 (4,8) |
| Women 35-44 | 70 (47,107) | 4 (3,6) | 6 (4,9) | 10 (7,15) | 19 (12,28) | 32 (21,49) |
| Women 45-54 | 272 (207,347) | 24 (18,31) | 38 (29,48) | 50 (38,64) | 63 (48,81) | 96 (74,123) |
| Women 55-64 | 794 (617,1009) | 86 (67,110) | 111 (86,140) | 147 (115,187) | 192 (149,244) | 258 (200,328) |
| Women 65-74 | 1732 (1351,2207) | 236 (184,301) | 275 (215,351) | 343 (268,438) | 389 (303,496) | 488 (380,622) |
| Women 75-84 | 3699 (2866,4678) | 612 (474,774) | 734 (568,928) | 758 (587,958) | 803 (622,1015) | 793 (614,1003) |
| Women 85+ | 6853 (5345,8684) | 1314 (1025,1665) | 1522 (1187,1929) | 1540 (1201,1952) | 1373 (1071,1740) | 1103 (861,1398) |
| *Total* | *37969 (30628,46522)* | *6558 (5297,8021)* | *7612 (6145,9316)* | *7999 (6454,9798)* | *7888 (6357,9677)* | *7911 (6374,9711)* |

CHD, Coronary Heart Disease; IMD, Index of Multiple Deprivation; BAPC, Bayesian Age Period Cohort; IMDQ, Index of Multiple Deprivation Quintile

*In the probabilistic analysis, 0 (0, 0) deaths were replaced with 1 (0, 1) death in order to fit a Pert distribution.

**References**

1. NatCen Social Research, University College London, Department of Epidemiology and Public Health (2016 ) Health Survey for England, 2014, 2nd Edition ed.: UK Data Service.

2. HM Revenue & Customs. UK Trade Statistics. <https://www.uktradeinfo.com/> (accessed February 2017

3. Department for Environment Food & Rural Affairs (DEFRA) (2017) *Agriculture in the United Kingdom 2016.*

4. WTO. Tariff Analysis Online. <http://tao.wto.org/default.aspx> (accessed February 2017

5. HM Government. Trade Tariff. <https://www.gov.uk/trade-tariff/sections> (accessed February 2017

6. European Commission (2016) *EU Agricultural Outlook. Prospects for EU agricultural markets and income 2016-2026*.

7. Swinbank A (2017) *World trade rules and the policy options for British agriculture post-Brexit*. UK Trade Policy Observatory.

8. Van Berkum S, Jongeneel R, Vrolijk H *et al.* (2016) *Implications of a UK exit from the EU for British agriculture*. no. 9462577730. LEI Wageningen UR.

9. British Soft Drinks Association. (2017) Making it Happen. Annual report 2017. <http://www.britishsoftdrinks.com/write/MediaUploads/Publications/BSDA_Drinks_Report_2017.pdf>

10. (2015) Market research provider, Euromonitor International. [www.euromonitor.com](http://www.euromonitor.com)

11. Office for Budget Responsibility (2016) Economic and fiscal outlook - March 2016.

12. Guzman Castillo M, Gillespie DOS, Allen K *et al.* (2014) Future Declines of Coronary Heart Disease Mortality in England and Wales Could Counter the Burden of Population Ageing. *PloS one* **9**, e99482.

13. Office for National Statistics (ONS) Number of registered deaths by sex, cause, year, the Adjusted Index of Multiple Deprivation 2010 quintiles (fifths) of Lower Super Output Areas and age group, England, 2002 to 2013.

14. Office for National Statistics Deaths registered in England and Wales: 2013.

15. Department for Environment Food & Rural Affairs (DEFRA) (2017) Family food datasets. <https://www.gov.uk/government/statistical-data-sets/family-food-datasets>

16. Afshin A, Peñalvo JL, Del Gobbo L *et al.* (2017) The prospective impact of food pricing on improving dietary consumption: A systematic review and meta-analysis. *PloS one* **12**, e0172277.

17. Colchero MA, Popkin BM, Rivera JA *et al.* (2016) Beverage purchases from stores in Mexico under the excise tax on sugar sweetened beverages: observational study. *BMJ* **352**, h6704.

18. NatCen Social Research, MRC Human Nutrition Research, University College London Medical School (2015 ) National Diet and Nutrition Survey Years 1-4, 2008/09-2011/12, 7th Edition ed.: UK Data Service.

19. Micha R, Peñalvo JL, Cudhea F *et al.* (2017) Association between dietary factors and mortality from heart disease, stroke, and type 2 diabetes in the united states. *JAMA* **317**, 912-924.

20. Allen K, Pearson-Stuttard J, Hooton W *et al.* (2015) Potential of trans fats policies to reduce socioeconomic inequalities in mortality from coronary heart disease in England: cost effectiveness modelling study. *BMJ* **351**, h4583.
